# Development and validation of an Eating Disorders Symptom Impact Scale (EDSIS) for carers of people with eating disorders

**DOI:** 10.1186/1477-7525-6-28

**Published:** 2008-04-21

**Authors:** Ana R Sepulveda, Jenna Whitney, Matthew Hankins, Janet Treasure

**Affiliations:** 1 Department of Psychological Medicine, King's College of London, Institute of Psychiatry, London, UK; 2 Department of Psychology, King's College of London, Institute of Psychiatry, London, UK; 3 Division of Primary Care & Public Health, Brighton & Sussex Medical School, University of Brighton, Falmer, UK

## Abstract

**Background:**

Family members of relatives with eating disorders experience high levels of distress due to the difficulties in their care giving role. However no measures have been developed to measure the specific impact that an individual with an eating disorder has on family life. The aim of this study was to develop a measure to assess the specific caregiving burden of both anorexia nervosa and bulimia nervosa. A secondary aim was to examine whether this measure was sensitive to change.

**Methods:**

A new scale, the Eating Disorders Symptom Impact Scale (EDSIS), was generated by a panel of clinicians and researchers based upon quantitative and qualitative work with carers and reviewed by a panel of "expert carers". A cross-sectional study was conducted among carers of relatives with an eating disorder to examine the properties of the new scale. In addition, participants from an ongoing pre-and-post design study completed several self-report questionnaires to assess the sensitivity of the EDSIS to change.

**Results:**

A sample of 196 carers of relatives with an eating disorder aged 25–68 compted the scale. A 24-item EDSIS scale was derived with four factors: nutrition, guilt, dysregulated behaviour and social isolation. These explained 58.4% of the variance in carer distress. Reliability was acceptable (Cronbach's alpha ranged from 0.84 to 0.90). The convergent validity of the EDSIS subscales was moderately supported by correlations with a general caregiving measure (Experience of Caregiving Inventory (ECI), r = 0.42 to 0.60), psychological distress (General Health Questionnaire (GHQ-12), r = 0.33) and perceived functioning of the relative (Children Global Assessment Scale (CGAS), r = -30). A sample of 57 primary caregivers completed pre-post intervention assessments and the overall scale (t = 2.3, p < 0.05) and guilt subscale (t = 3.4, p < 0.01) were sensitive to change following a skills training workshop.

**Conclusion:**

The EDSIS instrument has good psychometric properties and may be of value to assess the impact of eating disorder symptoms on family members. It may be of value to highlight carers' needs and to monitor the effectiveness of family based interventions.

## Background

Caring for individuals with a mental disorder can produce difficulties and stress for family members [1-3]. Caregiver burden refers to the physical, emotional, and social problems associated with caregiving [4]. Recently, the term "burden" has been re-defined to refer to carers' subjective and objective perceptions of the caregiving experience [5]. Measures to capture these concepts have been developed [6,7]. Burden is associated with psychological distress [8] and depressive symptomatology [9]. These measures highlight the relevance of caregiver burden in influencing the caregiver's life.

A review of sixteen measures of caregiver burden in relatives of schizophrenic patients was conducted by Reine and colleagues [10]. The most reliable validated instruments were the Perceived Family Burden Scale (PFBS), the Involvement Evaluation Questionnaire (IEQ) and the Experience of Caregiving Inventory (ECI). The latter instrument has been used to measure carer burden in people with anorexia nervosa (AN) [11,11,12] and bulimia nervosa (BN) [13]. However, although a rich literature describing the carers' experience from qualitative studies is available, [14-23] no specific measures have been developed to measure the impact that an individual with an eating disorder has on family life.

In addition, some of the themes described in the qualitative work with carers of people with eating disorders are similar to those found in other forms of psychiatric illness such as dependency, loss and a negative impact on work or finances. Other themes are more specific to eating disorders, for example, fear related to the dangers to physical health (i.e. low weight or vomiting) that the disorders pose for the sufferer, parental guilt concerning the notion that their action may have caused the illness, isolation resulting from the avoidance of eating socially. These aspects increase the concern about the possibility of long-term dependency as a result of the sufferer having lost opportunities and life experience. Other topics are related to the wide range of difficult situations at home resulting from the direct impact of eating disorder symptoms (i.e. difficulties with blocked drains, plumbing) or behaviours (i.e. stealing) on family life. Additionally, carers may be consumed with and concerned about the impact the strain involved in caring for their sufferer has on their own or other family member's health. Moreover, living with or looking after a person with an eating disorder generates an ample variety of contradictory emotions such as aggression crying, sadness, frustration, guilt and self-blame.

The primary aim of this study was to develop a measure to assess the specific caregiving burden of both anorexia nervosa and bulimia nervosa. The secondary aim was to examine whether the dimensions measured by this instrument might be amenable to change following a short intervention for carers.

## Methods

### Participants and procedure

Carers were recruited from the Eating Disorders Service of the South London and Maudsley Hospital (SLaM) from the National Health Service (NHS) Trust (n = 96) and from a Volunteer Database (n = 100) compiled by the eating disorder research unit. Carers on the research Volunteer Database were contacted by post and given an information sheet about the study and questionnaires with a freepost return facility. These participants were recruited over a period of two months. The carers from the Eating Disorder service were given the information regarding the assessment instruments at the same time they were offered the opportunity to participate in a Collaborative Care Workshop study. These participants were recruited over a year. To be eligible for the study, the carer had to be either living with, or directly involved in the care of a person with an eating disorder. The questionnaires from 190 relatives were included. The exclusion criterion regarding incomplete questionnaires was set at three or more incomplete items. Six questionnaires from the Volunteer Database were excluded. Ethical committee approval was granted for the study (Ref. No. 238/04).

### Volunteer Database

Parents on the Volunteer Database maintained by the Eating Disorder Unit (EDU) at the Institute of Psychiatry (IoP) and Maudsley Hospital are continually recruited from several sources, such as through a number of specialist services, via our annual newsletter which is widely distributed, and through other carer organisations such as Beat (*Beat *is a national charity based on UK providing support and help for people with eating disorders and their families). Carers are included in this database if they caring for a relative with an eating disorder. These carers are sent an information pack with general information about our ongoing research, the annual EDU newsletter and a short demographic form. For this study, the EDU newsletter and a flyer explained details about the study that was being offered. This post included an information sheet and a consent form as well as a new questionnaire and a short demographic form. At the time of recruitment for this project, the Volunteer Database consisted of 197 carers. One hundred carers completed the questionnaire and the demographic questions (a response rate of 50.1%) from the database. These carers were not offered incentives for participating.

### Participants from a Skills Intervention

In addition, the Collaborative Care Workshop study was also advertised on the website of the EDU and through the EDU newsletter sent to the carers on the Volunteer Database. Likewise, the study was also advertised through specialised eating disorder services from SLaM. A total of 30 newly recruited carers (15.2% response rate) from the Volunteer Database took part in this project. This sample was self-selected and we are unable to accurately comment on their reasons for non-participation nor how many from the SlaM eating disorder services refused to participate over the recruitment stage. The participants from this ongoing study completed a pack of self-report questionnaires (GHQ-12, ECI, CGAS described below). The aim of the carer workshops was to provide information and skills training for carers of people with eating disorders in order to improve their coping strategies and reduce the levels of difficulties and distress. The content of the intervention has been described previously[24,25]. A total of six, two hours long, workshops were held two evenings each month over three months. The results from a previous pilot study suggested that this skills based workshop was effective in reducing distress and caregiving burden. Changes in carers were maintained over 3 months and the content and method used in the workshops had a good acceptability amongst the carergivers [11].

The questionnaires mentioned above were also used as validity measures for the EDSIS as they assess general aspects of caregiving and psychological morbidity. The analyses in the validation described below was used the final version of EDSIS.

### The development of the Eating Disorder Symptom Impact Scale (EDSIS)

The items in the Eating Disorder Symptom Impact Scale (EDSIS) attempted to measure carers' appraisals of the personal impact that the eating disorder symptoms and behaviours of their ill relative had on their own well-being. They were generated by a panel of clinicians and researchers based on quantitative and qualitative work with carers and reviewed by a panel of "expert carers" [12,16,26,27]. The panel was made up of a psychiatrist (one of the authors of this paper), a clinical nurse leader and a social worker currently working at the South London and Maudsley Trust (SLaM) as well as five PhD research psychologists (of who two are also co-authors of this paper). All of the panel members were working at the Eating Disorder Unit at the time of the study. The expert carers (four mothers of daughters with eating disorders) were chosen for their previous experience in dealing with parents caring for a relative with an eating disorder. These four mothers have run carer support groups in the London region and have also collaborated closely with the EDU in previous research.

Several items were generated by transcripts from recordings of family interviews as part of a qualitative thesis developed at the EDU [12,16,17]. Following several in-depth discussions by the panel, a total of 30 statements were established based on criteria of clarity, relevance and significance for field-testing using a 5-point Likert-type scale (0 = never, 1 = rarely, 2 = sometimes, 3 = often, 4 = nearly always). Responses indicating the impact upon the carer within the previous month are shown in Appendix 1.

### Assessment Measures

#### Clinical and demographic Assessment

The participants reported the eating disorder symptoms and history of their cared-one in addition to providing demographic information for themselves and their cared-one.

#### General Health Questionnaire (GHQ-12) [GHQ-12;[28]]

The GHQ-12 was used to measure carers' level of psychological distress. Each item is rated on a 4-point scale with scores ranging from 0–36. Higher scores indicate increased psychological distress. Cronbach's alpha was 0.92.

#### Global Assessment Scale (GAS) [29]

The GAS measures the global severity of psychiatric illness and social disability. It has been adapted for children (CGAS) [30] and some further minor modifications were made in some terms to adjust it to an eating disorders context. The scale comprises ten equal-point intervals which describe general behavioural functioning on a hypothetical continuum of mental illness (range 1–100 where 100 is the healthiest).

#### Experience of Caregiving Inventory (ECI) [31]

The ECI measures the experience of caring for an individual with a severe mental illness. It has 66 items grouped in eight negative scales (Difficult Behaviours, Negative Symptoms, Stigma, Problems with Services, Effects on Family, Need to Backup, Dependency and Loss) with 52 items and two positive scales (Positive Personal Experiences and Good Relationship with the Patient) with 14 items. Higher scores indicate greater severity. Each scale has been reported to have satisfactory reliability (Cronbach alpha coefficient between 0.74 and 0.91 [31]). The reliability of the ECI was also estimated in our study: Cronbach's alpha was 0.93 for the total scale (66 items), 0.85 for the Positive scale and 0.94 for the Negative scale. Reliability ranged between 0.70 and 0.87 for the ten ECI dimensions.

### Statistical analysis

Individual missing values were replaced with the mean of same-gender carers when a maximum of three items were incomplete (n = 7). A series of analyses were conducted to test the psychometric properties of the EDSIS scale:

#### Principal Components Analysis

An exploratory factor analysis was performed using the principal component extraction method with Varimax rotation using SPSS.12. Eigenvalues of greater than one were used to select items for each of the domains (if loadings exceeded 0.40). A confirmatory factor analysis was then conducted. The KMO measure of sampling adequacy and Barlett's Test of Sphericity are reported for assessing factorability of the data.

#### Reliability

Scale reliability was assessed using Cronbach's alpha. Item-total and inter-item Pearson correlations were also calculated.

#### Validity

Convergent validity was examined using cross-sectional data to examine the strength of association between subscale scores of the final version of the EDSIS and the general aspects of caregiving (ECI), psychological distress (GHQ-12) and the global severity of the illness perceived by the carer (CGAS), using Pearson correlations. We expected a highest correlation with the negative dimension of the ECI. The validity was also explored by examining the association between type of diagnosis, patient's symptomatology and the EDSIS subscales.

#### Responsiveness

Student's *t*-test was used to assess measured change following the workshop intervention for carers. Effect sizes were calculated using Cohen's *d *to indicate the magnitude pre and post differences. The guidelines for interpreting this value (*d*) are: < 0.4 = small effect, >= 0.4 = moderate effect, >= 0.75 = large effect [32].

## Results

### Demographic Variables

Data from 190 carers was included in the validation study, 139 (73.2%) females and 47 (24.7%) males (n = 4, unspecified gender). The mean age of the carers was 51.1 years (SD = 8.6; range: 25–68). The patient group consisted of six males (7%) and 150 females (93%) with a mean age of 23.5 years (SD = 8.3; range: 9–54). A primary caregiver was defined as the individual who spent the most time with the patient. Thirty-four secondary caregivers were included reporting on the same patient. The clinical and demographic information is shown in Table 1.

**Table 1:** Demographic details of carers and patients

	**N**	**%**	**Mean (S.D.)**
*Carers*	190		

Age	-	-	51.1 (8.6)
Sex			
Male	47	24.7	
Female	139	73.1	
Marital status			
Married	127	78.0	
Not married	36	21.0	
Highest education level			
School Level	66	40.5	
Degree/Diploma level	79	48.4	
Employment status			
Full/Part time	107	56.3	
Not employed	52	27.3	
Relationship with sufferer			
Parents	129	89.6	
Husband/Partner	9	6.3	
Sibling	5	3.5	
Friend	1	0.7	
Living with patient*			
Yes	75	40.0	
No	20	10.5	
Amount of contact with patient per week*			
< 21 hours	16	30.0	
> 21 hours	36	70.0	

*Patient*	156		

Age	-	-	23.5 (8.3)
Sex			
Male	6	7.0	
Female	150	93.0	
Age of onset	-	-	15.5 (5)
Age when first diagnosed	-	-	18 (8)
Diagnosis (carers' report)*			
Anorexia	67	76.13	
Bulimia	21	23.86	

Note.*These questions were not included in the questionnaire given to the carers included in the Volunteer Database

### Volunteer Database

One hundred carers completed the new questionnaire from 197 carers. There were no differences in carers' demographic questions, such as education or marital status (p > 0.05) nor for patients' variables, such as 'type of diagnosis' or 'currently receiving treatment' (p > 0.05), between the participants who completed the questionnaire compared with those that did not complete.

### Intervention Sample

Ninety-six carers participated in the intervention program and measures were obtained from 66 carers (68.8%) following the intervention. Thirty participants that were secondary caregivers from the same relative were not included due to the problem of lack of independence for the pre-and-post analysis. A total of 57 primary caregivers had pre and post treatment assessments that were used for the data analysis (n = 57). Nine carers did not complete the post-intervention assessment. Fifty-three (93%) were females and four (7%) were male. The average age of the carers was 51.4 years (SD = 7.3). Thirty-seven (67%) worked full or part time. Eighteen (32%) of the carers were educated up to secondary level and 36 (62%) were educated to higher education; no information was given from the rest of carers (6%). Fifty-four carers (95%) were parents and three (5%) were sisters or friends. Forty-four carers (79%) were currently living with the patient. Thirty-six carers (70%) reported more than 21 hours of contact with the patient per week.

### Principal components analysis

Principal components analysis (PCA) revealed a six-factor structure explaining 38.0% of the total variance. A further analysis excluding items with factor loadings smaller than 0.4 or with equal loading on more than one factor revealed a four-factor solution with 24 items accounting for 58.4% of the variance of 24 items. The Kaiser-Meyer-Oklin (KMO) measure of sampling adequacy was 0.85, exceeding the recommended 0.6 and Barlett's Test of Sphericity reached statistical significance (p < 0.01), supporting the factorability of the correlation matrix. These factors were interpreted as themes of nutrition, guilt, dysregulated behaviour and social isolation. Table 2 shows the item loadings, variance explained, item-total correlations and reliabilities for these four subscales.

**Table 2:** Factor matrix following varimax rotation for EDSIS scale

**Item**	**Factor Loadings**				**Item-scale correlation**	**Communality**
	
	**1**	**2**	**3**	**4**		
*Factor 1:Nutrition (Cronbach alpha 0.84)*						
Did you experience difficulties preparing meals	**0.68**	0.08	0.15	0.05	0.60	0.53
Were there arguments or tension during mealtimes	**0.68**	-0.05	0.13	0.17	0.61	0.69
Did you have to turn up the heat due to her/him feeling cold	**0.66**	0.11	0.00	-0.02	0.51	0.51
Did you notice or think about how the illness was affecting her/him mentally	**0.66**	0.21	0.17	0.20	0.62	0.68
Were there arguments with other family members about how to handle mealtimes	**0.65**	0.04	-0.03	0.21	0.55	0.65
Did you notice or think about how the illness was affecting her/him physically	**0.64**	0.14	0.08	0.11	0.56	0.68
Did you check on her to ensure that she/he was okay	**0.64**	0.19	0.09	0.23	0.60	0.60
Did you spend long period of time shopping for food	**0.55**	0.14	0.18	0.02	046	0.61
*Factor 2: Guilt (Cronbach alpha 0.89)*						
Feeling that I have let her/him down	0.17	**0.89**	0.08	0.17	0.85	0.84
Feeling that there could have been something that I should have done	0.20	**0.88**	0.12	0.11	0.86	0.85
Thinking about where I went wrong	0.24	**0.85**	0.13	0.03	0.80	0.80
Feeling that I should have noticed it before it became so bad	-0.02	**0.79**	0.08	0.11	0.64	0.64
Thinking that perhaps I was not strict enough	0.16	**0.65**	0.18	0/.13	0.54	0.49
*Factor 3:Dysregulated Behaviour (Cronbach alpha 0.82)*						
Did you have difficulties with blocked drains, plumbing	-0.10	0.11	**0.75**	-0.03	0.56	0.63
Lying/stealing	0.15	0.13	**0.72**	0.06	0.61	0.64
Out of control temper	0.12	0.09	**0.72**	0.27	0.64	0.84
Physically and/or verbally aggressive	0.15	0.10	**0.70**	0.28	0.65	0.84
Did food disappear from the cupboards	0.16	-0.03	**0.61**	-0.37	0.43	0.66
Were there bad smells and poor hygiene in the bathroom	0.11	0.14	**0.58**	-0.09	0.47	0.73
Controlling/manipulative	0.34	0.06	**0.55**	0.23	0.50	0.61
*/Factor 4: Social isolation (Cronbach alpha 0.86)*						
How your friends/relatives have stopped visiting	0.15	0.20	-0.05	**0.84**	0.75	0.75
Losing your friends	0.06	0.12	0.07	**0.77**	0.64	0.71
Cancelling or refusing plans to see friends or relatives	0.37	0.14	0.09	**0.77**	0.77	0.75
Feeling unable to go out for evenings, weekends or on holiday	0.32	0.09	0.19	**0.70**	0.70	0.67
**Eigenvalue**	3.99	3.66	3.36	2.99		
**Percentage Variance explained**	16.6	15.2	14.0	12.5		
Cumulative percentage variance explained	16.6	31.9	45.9	**58.4**		

Note. Bold values show on-factor loadings.

Additional file 1 shows the 30-original statements chosen for the EDSIS. The six following items 5, 6, 12, 18, 22 and 25 were deleted after the principal components analysis.

### Scoring the EDSIS

For the main analysis, total rating for each factor was computed by adding the scores of the items belonging to a specific domain (nutrition, guilt, dysregulated behaviour and social isolation). The score range for each item was from 0 to 4: scale score ranges for subscale dimensions therefore varied with the number of items of the subscale. Additionally, a total score was calculated in two ways a) by summing the scores of all the items in order to obtain an overall score of caregiving burden specific to eating disorders and, b) by computing a total score by adding the mean domain scores of each scale. As the two methods gave scores which correlated >0.9, we concluded that the EDSIS total score would be obtained by summing the unweighted scores of all the items: the total scale is therefore scored from 0 to 96. A higher score means more negative appraisals on specific aspects of caregiving.

### Scale reliability

The Cronbach's alphas for the subscales were 0.89 (Nutrition), 0.84 (Guilt), 0.82 (Dysregulated Behaviours), and 0.86 (Social Isolation). Cronbach's alpha for the total instrument was excellent (α = 0.90).

### Convergent Validity

Convergent validity was determined through correlations between EDSIS and the ECI-negative (8 subscales), the ECI-positive (2 subscales), the GHQ-12 questionnaire and CGAS scale. The results are illustrated in Table 3. All dimensions of the Eating Disorders Symptom Impact Scale were related to the overall general negative burden measured by the Experience of Caregiving Inventory with correlations ranging from 0.42 to 0.60 (p < 0.05). There was a significant relationship between the perceived functioning of the eating disordered individual (CGAS) and social functioning of carers (EDSIS) and the total score of the scale (EDSIS). A significant positive correlation size was also found between EDSIS and GHQ-12.

**Table 3:** Correlations between the EDSIS subscales scores and ECI-negative, ECI-positive, GHQ and CGAS (N = 96)

**ED subscales**	**Dysreg B.**	**Guilt**	**Isolation**	**EDSIS**	**ECI-negative**	**ECI-positive**	**GHQ-12**	**CGAS**
***Nutrition***	0.27*	0.28**	0.37**	0.74**	0.48**	0.50	0.18	-0.27*
***Dysregulated Behaviour ***	-	0.16	.082	0.64**	0.45**	0.08	0.23*	-0.18
***Guilt***	-	-	0.21*	0.58**	0.42**	0.09	0.24*	-0.11
***Social Isolation***	-	-	-	0.55**	0.60**	-0.15	0.33**	-0.30*
***EDSIS Total score (24 items)***	-	-	-	-	0.71**	0.20	0.32**	-0.36**

Note. ** Correlation is significant at the 0.01 level (2-tailed).

* Correlation is significant at the 0.05 level (2-tailed).

### The Relationship between the Eating Disorders Symptom Impact Scale (EDSIS) subscales and clinical and demographic variables

As demonstrated by Table 3, correlations between the subscales scores were low, but most of them were significant, with correlations ranging between 0.21 and 0.37 (p < 0.05). Correlations between some patient characteristics such as duration of the illness, average hours of contact, total time spent at the hospital or currently receiving treatment, and the scales of EDSIS, ECI-negative and GHQ-12 were low, and most were non significant. Significant correlations were found between type of diagnosis and Dysregulated Behaviour subscale (N = 88, *r *= 0.36, p = 0.03) with higher scores for those with bulimia nervosa; the level of underweight correlated with the Nutrition, Guilt and Social Isolation subscales (N = 90, *r *= 0.23,0.21 and 0.26, p < 0.05, respectively); and vomiting behaviour and binging correlated with the Dysregulated Behaviour subscale (N = 91, *r *= 0.44 and 0.53, p < 0.01).

The specific difficulties measured with the Eating Disorders Symptom Impact Scale (EDSIS) and the general caregiving difficulties (ECI) by diagnosis and cohabitation status (ECI) are shown in Table 4. Carers of patients with bulimia nervosa experienced higher levels of general and specific caregiving difficulties than those with anorexia nervosa. In particular, they endured over twice as much dysregulated behaviour as carers of people with anorexia nervosa (p < 0.05;*d *= 1.14).

**Table 4:** Carers' EDSIS scores by eating disorder diagnosis and living situation.

**Variables**	**Nutrition**	**Dysregulated Behaviour**	**Guilt**	**Isolation**	**EDSIS**	**Positive ECI**	**Negative ECI**
**AN carers (N = 67) Mean (SD)**	17.7 (6.2)	6.5 (4.7)**	11.5 (4.5)	6.2 (4)	41 (12.6)	28 (7)	96 (26)*
**BN carers (N = 21) Mean (SD)**	17.5 (5.9)	12 (6.5)**	12.3 (5.2)	5.3 (4.4)	47.2 (14)	29.9 (9.5)	114 (35)*
**Living with (N = 75) Mean (SD)**	17.7 (5.6)	8.6 (5.8)*	11.8 (4.7)	6.4 (4.3)	44.5 (13)*	28 (7.6)	102 (29)
**Living without (N = 18) Mean (SD)**	16.5 (7.3)	5.5 (4.5)*	11 (4.9)	5 (4)	36 (11.6)*	29.6 (6.8)	92.2 (28)

Note. * p < 0.05 ** p < 0.05,

In general, carers whose offspring were living away from home experienced lower levels of dysregulated behaviour and less specific difficulties related with the illness (EDSIS total score) (p < 0.05; *d *= 0.64 and 0.66, respectively).

### Responsiveness to change

The total score and the guilt subscale of the EDSIS showed an improvement after the workshop intervention with a moderate effect size (see Table 5). This paralleled reduction in negative aspects of caregiving (measured by ECI-negative) and carers' psychological distress (GHQ-12) and an improvement patient's functioning (CGAS) following the intervention.

**Table 5:** Workshop Intervention effect from pre- to post-intervention from EDSIS, ECI, GHQ-12 and CGAS scores

**Variables**	***N***	***Baseline (T_*1*_) Means (SD)***	***Post-interv (T_*2*_) Means (SD)***	***t***	***p***	***d***
**Eating Disorders Symptom Impact (EDSIS)**						
***Total score of combined scales (0–96)***	48	42.8 (13.8)	38.2 (16.2)	2.3	**0.027**	***0.31***
***Nutrition(0–32)***	49	16.6 (6.4)	15.2 (7.1)	1.4	0.18	*0.21*
***Dysregulated behaviour(0–28)***	50	8.1 (5.6)	7.7 (5.8)	0.56	0.57	*0.08*
***Guilt(0–20)***	49	12 (4.7)	9.4 (4.7)	3.4	**0.001**	***0.56***
***Social Isolation(0–16)***	50	6.3 (4.2)	5.5 (4.2)	1.5	0.13	*0.2*
**Experience of Caregiving Inventory (ECI)**						
***ECI-Positive(0–56)***	50	29.1 (7.5)	31.2 (7.7)	-1.8	0.07	*0.27*
***ECI-Negative(0–208)***	44	101.6 (26.7)	88.4 (31.8)	3.7	**0.001**	*0.45*
**General Health Questionnaire (GHQ-12)**						
***Total score(0–36)***	41	17.7 (13.3)	12.3 (16.7)	4.6	**0.001**	*0.36*
**Informant rating of Index Case (CGAS)**						
***Total score(0–100)***	33	56.8 (15.7)	62.8 (14.1)	-2.1	**0.04**	*0.41*

## Discussion

The primary aim of this study was to develop an Eating Disorders Symptom Impact Scale (EDSIS) to measure the specific caregiving difficulties for families of people with an eating disorder. Furthermore, this scale has been able to discriminate between differential interpersonal burden caused by caring for a relative with anorexia nervosa or by bulimia nervosa. We found that an instrument with 24 items and four factors: nutrition, dysregulated behaviour, guilt, and social isolation, encapsulated the specific difficulties encountered by such families.

The final instrument comprises two factors directly related to the specific problems caused by eating disorder symptoms and two factors more indirectly related to the symptoms but resulting from carers' personal reactions (guilt and social isolation) to the illness. The internal consistencies of the four factors were high above the standard of 0.70 set by Nunnally and Bernstein [33] for newly developed research tools. The convergent validity was examined comparing this specific caregiving instrument with the non specific experience of caregiving inventory (ECI). Smaller associations were found between the social isolation factor and carers' level of distress (GHQ-12) and a global measure of eating disorder symptomatology (CGAS).

The carers of people with bulimia nervosa experienced higher levels of general and specific caregiving difficulties than those with anorexia nervosa as measured by the total scale and subscales of the EDSIS. These carers endorsed twice as much dysregulated behaviour and general caregiving difficulties (p < 0.05). It is interesting to note that only 16 of the 21 patients with bulimia nervosa were currently living at home with the primary caregiver while 65 out of 67 patients with anorexia nervosa were currently living with their primary caregiver. The impact of dysregulated behaviour and the overall specific caregiving problems was reduced if the individual with an eating disorder was not living at home.

The secondary aim of this study was to examine if the dimensions measured by this instrument might be amenable to change. The total score and the score for the guilt subscale did decrease significantly following a brief group intervention. The psychoeducational content of the workshop and group approach (i.e., having multiple carers of people with eating disorders ranging from 12 to 16 carers per group) addressed unhelpful appraisal of guilt and blame. The failure to produce change on the other three factors (nutrition, dysregulated behaviour and social isolation) suggests that interventions need a greater focus on producing change in these domains. Thus, more time and effort should be focused on strategies aimed at increasing social connections for carers. Also a greater emphasis on teaching behavioural techniques, such as a functional analysis, to reduce eating symptoms (such as dysregulated behaviours and poor nutritional health) may be helpful. The ECI was not sensitive to change in the context of family interventions for schizophrenia [34,35] and it is hoped that the EDSIS measure will offer more potential as a measure of the effectiveness of interventions for carers.

There are some limitations that should be noted. Firstly, the results of this study require replication, ideally with a larger and more diverse sample of caregivers. Secondly, while information was gained about the measure's sensitivity to change before and after a carers' intervention, additional attention should also be given to assessing test-retest reliability, a property that was not assessed in the current paper. A third limitation was that characteristics of the illness were collected from a self-report survey completed by carers and not collected using diagnostic or standardised measures. Fourthly, carers were recruited to the volunteer database from a number of specialist services and via the magazine of the main voluntary user and carer organisation and the generalisability of these results are uncertain.

## Conclusion

There is currently no questionnaire designed specifically to measure the specific caregiving burden associated with eating disorder symptomatology. The Eating Disorder Symptom Impact Scale (EDSIS) has good psychometric properties and some clinical utility. This instrument may be of value to highlight the specific needs of families of people with eating disorders and to tailor family work to these areas.

## Abbreviations

EDSIS – Eating Disorder Symptom Impact Scale; GHQ-12 – General Health Questionnaire; ECI – Experience Caregiving Inventory; CGAS – Children Global Assessment Scale; AN – Anorexia Nervosa; BN – Bulimia Nervosa; M – Mean; SD – Standard Deviation; *d *– effect size; N – sample size; Dysre B. – Dysregulated Behaviour; SLAM – South London and Maudsley Hospital; EDU – Eating Disorder Unit; IoP – Institute of Psychiatry.

## Competing interests

The authors declare that they have no competing interests.

## Authors' contributions

ARS conceived and designed the study, oversaw all stages of data collection and analysis, and drafted the manuscript. JW conducted focus groups, provided clinical advice on design and did qualitative analysis for the items, and reviewed the manuscript. MH revised the data analysis, participated in consensus item selection processes and reviewed the manuscript. JT coordinated all stages of the study, gave feedback on design and reviewed the manuscript. All authors read and approved the final manuscript.

## Supplementary Material

Additional file 1 The 30-original statements for the Eating Disorders Symptom Impact Scale (EDSIS). The data provided illustrates the 30-items were selected as part of the first questionnaire and the instructions that were given to the carer participants. Click here for file
