# Supplementary material for: Development and validation of an Eating Disorders Symptom Impact Scale (EDSIS) for carers of people with eating disorders
Source: Health Qual Life Outcomes. 2008 Apr 21;6:28. doi: 10.1186/1477-7525-6-28 (PMC2365933; doi:10.1186/1477-7525-6-28)
Supplement: Additional file 1 — The 30-original statements for the Eating Disorders Symptom Impact Scale (EDSIS). The data provided illustrates the 30-items were selected as part of the first questionnaire and the instructions that were given to the carer participants. [file 1477-7525-6-28-S1.pdf]

Appendix A: The 30-original statements for the Eating Disorders Symptom Impact Scale (EDSIS)

Name:…………………………………...........Date……………………………………

The following pages contain a number of statements that commonly apply to persons who care for relatives or friends with an eating disorder. We would like you to read each one and decide how often it has applied to you over the **past one month**. It is important to note that there are no right or wrong answers. Also, it is best not to spend too long on any one statement. Your first reaction will usually provide the best answer.

**Items**	**Never**	**Rarely**	**Some-times**	**Often**	**Nearly Always**
-During the past month how often have you thought about:					
1. How your friends/relatives have stopped visiting……	0	1	2	3	4
2. Losing your friends………………………………….	0	1	2	3	4
3. Feeling unable to go out for evenings, weekends or on holiday……………………………………………...	0	1	2	3	4
4. Cancelling or refusing plans to see friends or relatives…………………..………………………...	0	1	2	3	4
*5. Sometimes feeling guilty, we are both adults and yet cannot work this out ……………………………….	0	1	2	3	4
*6. Feeling very sensitive sometimes when people are looking at her/him…………………………………	0	1	2	3	4
7. Feeling that I should have noticed it before it became so bad…………………………………………………	0	1	2	3	4
8. Feeling that I have let her/him down…………….	0	1	2	3	4
9. Feeling that there could have been something that I should have done…………………………………...	0	1	2	3	4
10. Thinking that perhaps I wasn't strict enough……	0	1	2	3	4
11. Thinking about where I went wrong……….……	0	1	2	3	4
*12. The effects on other members of the family as I cannot give them as much attention and this makes me feel guilty. ………………	0	1	2	3	4
13. Physically and/or verbally aggressive…………..	0	1	2	3	4
14. Controlling/manipulative………………………	0	1	2	3	4
15. Lying/stealing………………………………….	0	1	2	3	4
16. Out of control temper……………………...……	0	1	2	3	4
- When the sufferer was living with you at home during the past month, how often: (if the sufferer was not living at home with you during the past month, please refer to the last time she/he was living at home)					
17. Did you experience difficulties preparing meals (i.e. making separate meals for family members, not having correct ingredients)? ………………………………	0	1	2	3	4
*18. Did the sufferer refuse to eat with the family? …	0	1	2	3	4
19. Were there arguments with other family members about how to handle mealtimes? …………………	0	1	2	3	4
20. Were there arguments or tension during mealtimes?	0	1	2	3	4
21. Did food disappear from the cupboards? ………	0	1	2	3	4
*22. Did you spend large amounts of money shopping for food?	0	1	2	3	4
23. Did you spend long periods of time shopping for food? ……………………………………………	0	1	2	3	4
24. Did you have difficulties with blocked drains, plumbing? ………………………………………	0	1	2	3	4
*25. Were there arguments about the bathroom not being available for others? ……………………………….	0	1	2	3	4

26. Were there bad smells and hygiene in the bathroom?	0	1	2	3	4
27. Did you have to turn up the heat due to her/him feeling cold? ………………………………………	0	1	2	3	4
28. Did you check on her/him to ensure that she/he was "*okay*"? …...…………....…………………………..	0	1	2	3	4
29. Did you notice or think about how the illness was effecting her/him physically (i.e. see her/him fall, faint, struggle up the stairs)? ……………….……...……..	0	1	2	3	4
30. Did you notice or think about how the illness was effecting her/him mentally? ……………………….	0	1	2	3	4
